# Circulating prolactin level in Juvenile Systemic Lupus Erythematosus and its correlation with disease activity: a case control study

**DOI:** 10.1186/s12969-023-00915-7

**Published:** 2023-10-20

**Authors:** Hend Mehawed Soliman, Balsam Sherif Fahmy, Moataz Gamal Ali, Eman Shafik Shafie

**Affiliations:** 1 Pediatrics Department, Faculty of Medicine, Children`s Hospital, Kasr Alainy, Cairo University, Cairo, Egypt; 2 Clinical and Chemical Pathology Department, Faculty of Medicine, Kasr Alainy, Cairo University, Cairo, Egypt; 3 Pediatrics Department, Sheikh Zayed Specialized Hospital, Sheikh Zayed City, Egypt

**Keywords:** SLE, Prolactin, SLEDAI

## Abstract

**Background:**

The linkage between prolactin (PRL) and systemic lupus erythematosus (SLE) is still vague. Determination of serum levels of prolactin to reveal its role in patients with SLE is the aim of the study.

**Methods:**

This is a case-control study performed on 40 children with SLE and 40 age- and sex-matched controls. Cases were further subdivided according to disease activity into mild, moderate, and severe groups using the SLEDAI (Systemic Lupus Erythematosus Disease Activity Index) score. Serum prolactin levels were assayed by ELISA (enzyme-linked immunosorbent assay).

**Results:**

prolactin level was significantly higher in SLE patients (17.3 ± 6.6 µg/L) than in controls (13.5 ± 5.3 µg/L) (P value = 0.005). Although the prolactin level was highest in severe cases (19.3 ± 7.7 µg/L), followed by moderate cases (17.0 ± 5.3 µg/L), and lowest in mild cases (14.0 ± 6.2 µg/L), the variance between the 3 groups was not statistically significant (P value = 0.212). A significant positive correlation between prolactin level and SLEDAI score was detected (r = 0.368) (P value = 0.019). Hyperprolactinemia was found in 8 patients (20%) but not in controls; 4 out of 8 patients with hyperprolactinemia (50%) showed neurological manifestations compared to only 3 out of 32 patients with a normal prolactin level (9.4%) (P value = 0.007).

**Conclusion:**

A relationship between serum prolactin levels and juvenile SLE disease was detected. Neurological manifestations were more prevalent among SLE patients with hyperprolactinemia.

## Background

Prolactin (PRL) is a lactogenic hormone and an immunomodulator that enhances lymphocyte survival, stimulation, and proliferation. It is released by the anterior pituitary gland and extrapituitary sites as immune cells. Prolactin stimulates immune cells by binding to the prolactin receptor (PRL-R), which is a member of the hematopoietic cytokine receptor superfamily, allowing prolactin to have cytokine and endocrinological roles [1]. Earlier studies have shown that prolactin is implicated in autoimmunity and has a role in regulating both cell-mediated and humoral immune responses [2]. Also, it could influence the onset and severity of autoimmune illnesses like SLE and others [3, 4]. It was reported that serum prolactin levels were increased in SLE patients of both sexes, with an incidence of 20–30%, particularly in active disease [5]. Moreover, when bromocriptine is used to suppress the secretion of prolactin, it has a favourable outcome in some SLE patients [6]. There are conflicting results as regards the role of prolactin in SLE initiation and disease severity. Some studies found that prolactin level was not correlated with SLE disease activity and that prolactin level did not vary significantly when compared to control subjects [7–10]. Alternatively, other studies suggested that prolactin was associated with the involvement of major organs such as lupus nephritis and neuropsychiatric lupus [11, 12]. In our study, we tested for serum prolactin in juvenile SLE patients compared to the control group, aiming to reveal its role in disease pathogenicity and severity.

## Methods

### Ethical considerations

The present study has received approval from Cairo University’s Faculty of Medicine’s research ethics committee (approval number: MS-105-2019) and was carried out in line with the guidelines of the Helsinki Declaration of 1975. Prior to clinical data and sample collection, informed consent was obtained from patients’ guardians. Patients’ collected data were kept confidential.

### Study design and data collection

This is a case-control study performed on 40 children with SLE recruited from rheumatology wards in Cairo University Hospitals and age- and sex-matched healthy controls seeking routine checkups in outpatient clinics from January 2022 to June 2022. The inclusion criteria involved patients less than 16 years old of both genders diagnosed with SLE according to the American College of Rheumatology (ACR) criteria [13]. Cases were further subdivided according to disease activity using the Systemic Lupus Erythematosus Disease Activity Index (SELDAI) into absent disease activity (denoted by score 0–3), mild disease activity (denoted by score 4–8), moderate disease activity (denoted by score 8–12), and severe disease activity (denoted by score > 12) [14]. Exclusion criteria included endocrinopathies (e.g., hypothyroidism, prolactin-producing endocrinal tumours), creatinine > 2 mg/dl, and medications that alter the level of prolactin. Patients were classified as having neurological disorders, e.g., seizures; hematological disorders, e.g., leucopenia; immunological disorders, e.g., low complement levels; and renal disorders, e.g., proteinuria > 0.5 g/24 h, according to ACR criteria [13]. Hyperprolactinemia was identified as PRL levels ≥ 20 µg/L in males and > 25 µg/L in females [15]. Tanner staging was used in assessing pubertal development, and if any of the pubertal features appeared, e.g., axillary or pubic hair, the child was categorized as having pubertal features [16]. The fact of being descended from the same progenitor was used to define consanguinity. The presence of a family member with SLE disease was used to categorize patients as having a family history of SLE. Systolic and/or diastolic blood pressure in children that is above the 95th percentile for their age, gender, and height is referred to as hypertension [17]. Weight standard deviation scores (SDS), height SDS, and BMI SDS were assessed and plotted on appropriate centiles for age and sex [18]. Medications used for patient management include steroids, hydroxychloroquine (antimalarial), azathioprine, cyclophosphamide, cyclosporine, and mycophenolate mofetil. Intravenous methylprednisolone at a dose of 30 mg/kg to a maximum of 1 g (for 1–5 consecutive days) was initiated at diagnosis, followed by daily doses of glucocorticoids (0.5–2 mg/kg/day), and then tapered based on improvement in disease activity and response to treatment, with respect to improvement in laboratory parameters [19].

### Analysis

Blood samples were obtained between 9 and 11 a.m. and after resting for 10 min to avoid stress. Serum was taken following centrifugation and stored at -20 °C until prolactin assay by a commercially available enzyme-linked immunosorbent assay (ELISA) kit (DRG Diagnostics, Marburg, Germany). The following labs were recruited from files: serum creatinine, BUN (blood urea nitrogen), AST (aspartate transaminase), ALT (alanine transaminase), CBC (complete blood count), ESR (erythrocyte sedimentation rate) (normal values: ≤ 10–20 mm/hr for children to 12 years old, ≤ 15 and ≤ 20 mm/hr for males and females > 12 years, respectively), urine analysis, the ratio of albumin/creatinine in the urine, anti-double strand DNA (anti-dsDNA), anti-nuclear antibodies (ANA), and serum complements (C3, C4) (normal values: C3 levels: 88 to 201 mg/dl, C4 levels: 15 to 45 mg/dl) [20].

### Statistical analysis

Data were analyzed using Statistical Package for Social Sciences (SPSS) software (version 28.0). Quantitative data were expressed as mean ± SD (standard deviation) in addition to minimum and maximum range, compared by the independent t-test (two independent groups) and the ANOVA test (three independent groups). Qualitative data were expressed as numbers and percentages and compared by the Chi-square test and Fisher’s exact test. The Pearson correlation coefficient (r) was used for correlation testing. A P value of < 0.05 was considered significant.

## Results

Our study was composed of 40 SLE children and adolescents; 6 were males (15.0%) and 34 were females (85.0%), and 40 normal children and adolescents; 7 were males (17.5%) and 33 were female (82.5%). Both groups were age- and sex-matched (P values 0.095 and 0.762, respectively) (Table 1).

Also, no statistically significant variance between both groups exists regarding consanguinity (P value = 0.075). While family history for SLE was more frequent in cases than control, the variance was not statistically significant (P value = 0.055) (Table 1).

The main clinical manifestations in our study were arthritis (67%) followed by a malar rash (65%), while the less frequent manifestations were renal disorders (57.5%), hematological disorders (47.5%), neurological disorders (17.5%), and discoid rash (5%).

Regarding the anthropometric measures, SLE patients had significantly lower weight SDS and body mass index SDS (BMI) (P values 0.012 and 0.043, respectively). Moreover, hypertension was significantly more prevalent among cases than controls (P value = 0.002) (Table 1).

As regards the laboratory data, cases had significantly higher PRL and ESR (P values 0.005 and < 0.001, respectively). Moreover, 75% of cases showed positive ADNA, and 95% showed positive ANA (Table 1).

**Table 1:** Demographic, anthropometric and laboratory findings of SLE patients and controls

Variables			SLE cases (N = 40)	Control (N = 40)	P-value
Age (years)		Mean ± SD	11.5 ± 2.1	10.7 ± 1.9	^^^0.095
		Range	6.4–15.3	7.8–15.5	
Sex, N (%)	Male		6 (15.0%)	7 (17.5%)	^#^0.762
	Female		34 (85.0%)	33 (82.5%)	
Consanguinity, N (%)			14 (35.0%)	7 (17.5%)	^#^0.075
Family history of SLE, N (%)			5 (12.5%)	0 (0.0%)	^§ 0^.055
Weight SDS, mean ± SD			-0.3 ± 1.1	0.3 ± 0.9	^^^0.012*
Height SDS, mean ± SD			-0.2 ± 1.0	0.2 ± 1.0	^^^0.070
BMI SDS, mean ± SD			-0.2 ± 1.1	0.2 ± 0.9	^^^0.043*
Hypertension, N (%)			9 (22.0%)	0 (0.0%)	^§ 0^.002*
Puberty features, N (%)			22 (55.0%)	32 (80.0%)	^#^0.017*
Platelets (x10^3^/ml), mean ± SD			290.5 ± 100.6	302.9 ± 93.4	^^^0.567
ESR (mm/hr), mean ± SD			39.4 ± 27.0	7.8 ± 2.8	^^^<0.001*
C3 (mg/dl), mean ± SD			97.4 ± 64.7	105.8 ± 26.2	^^^0.450
C4 (mg/dl), mean ± SD			22.0 ± 22.3	24.3 ± 6.6	^^^0.538
Positive ADNA, N (%)			30 (75.0%)	0 (0.0%)	^#^<0.001*
Positive ANA, N (%)			38 (95.0%)	0 (0.0%)	^#^<0.001*
Prolactin (µg/L), mean ± SD			17.3 ± 6.6	13.5 ± 5.3	^^^0.005*
SLEDAI score, mean ± SD			11.74 ± 3.9		

^Independent t-test. #Chi square test. §Fisher’s Exact test

*P value < 0.05

BMI: body mass index, ESR: erythrocyte sedimentation rate, ADNA: anti-double stranded DNA, ANA: antinuclear antibodies, SDS: standard deviation scores

**Fig. 1:**
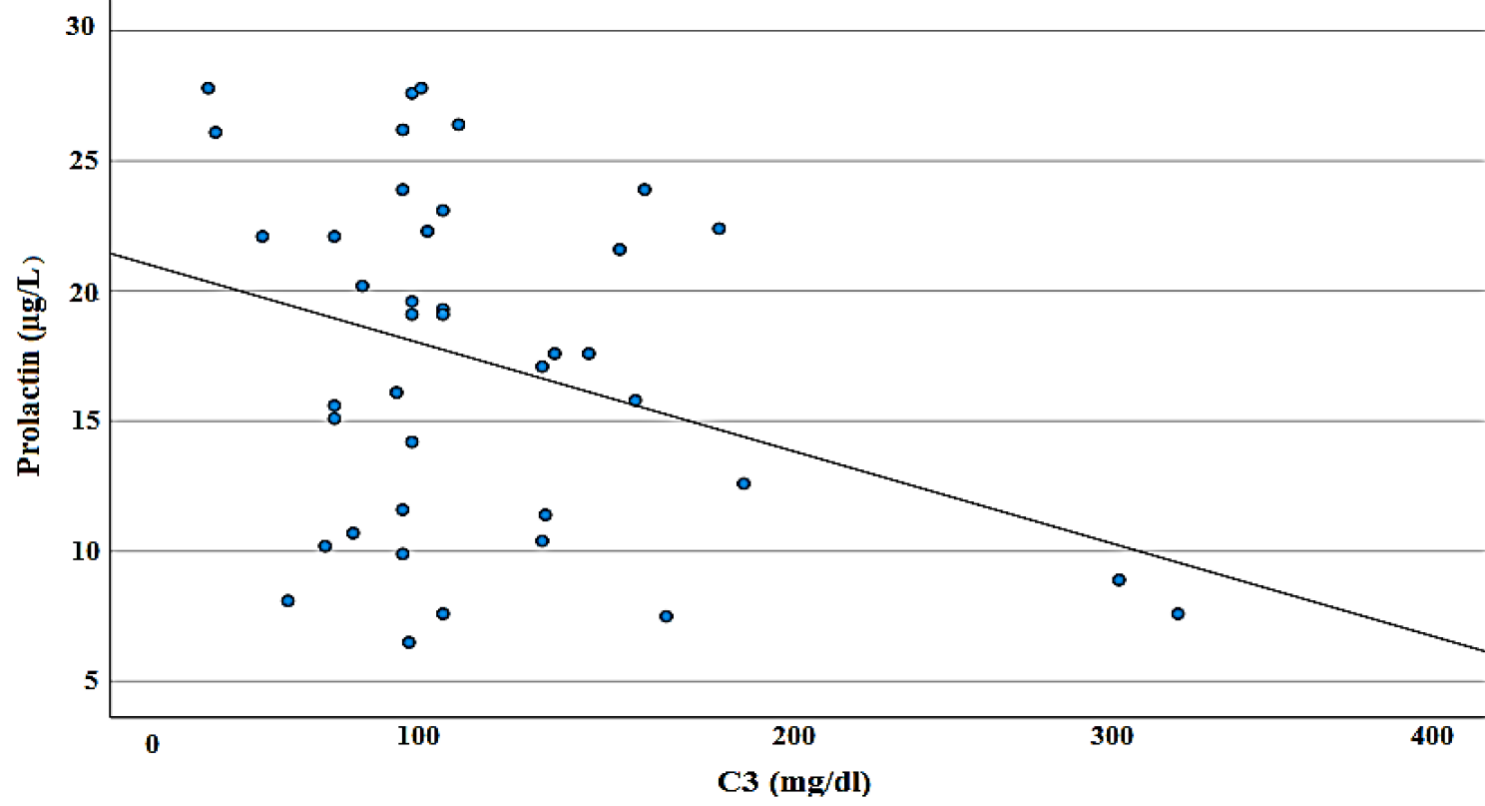
Scatter plot displaying the correlation between prolactin level and the C3 level among the studied patients (N = 40)

**Fig. 2:**
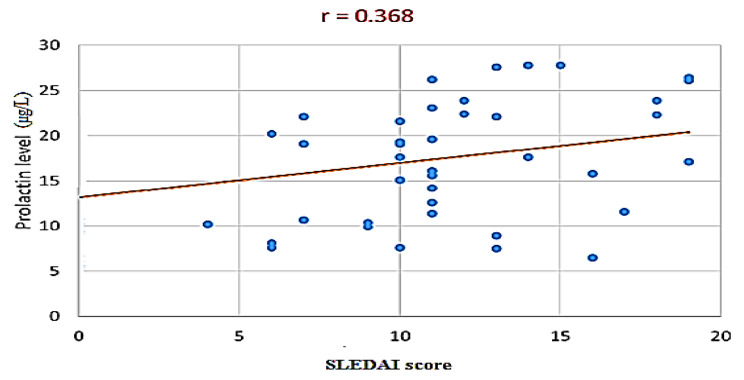
Scatter plot displaying the correlation between prolactin level and the SLEDAI score among the studied patients (N = 40)

Moreover, the Pearson correlation test revealed a significant negative correlation between prolactin and C3 in the SLE group (r = -0.339, P value = 0.032) (Table 2; Fig. 1). Additionally, the correlation between prolactin level and disease activity evaluated by the SLEDAI score showed a statistically significant positive correlation (r = 0.368, P value = 0.019) (Table 2; Fig. 2).

**Table 2:** Correlation between serum prolactin level and some anthropometric and laboratory findings

Variables	SLE cases (N = 40)		Control (N = 40)	
	r	P-value	r	P-value
Age	0.122	0.452	-0.035	0.831
Weight (SDS)	0.099	0.544	-0.231	0.152
Height (SDS)	0.205	0.204	-0.100	0.539
BMI (sds)	-0.091	0.576	-0.182	0.262
Platelets (x10^3^/ml)	-0.080	0.625	0.102	0.533
ESR (mm/hr)	-0.069	0.671	0.105	0.518
C3 (mg/dl)	-0.339	0.032*	-0.011	0.949
C4 (mg/dl)	-0.278	0.082	-0.114	0.484
SLE activity score (SELDAI)	0.368	0.019*		

r: Correlation coefficient. *P value < 0.05. BMI: body mass index, ESR: erythrocyte sedimentation rate, ADNA: anti-double stranded DNA, ANA: antinuclear antibodies, SDS: standard deviation scores

In this study, the categorization of SLE patients according to disease activity showed that 17.5% showed mild disease activity, 45% showed moderate disease activity, and 37.5% had severe disease activity. No statistically significant difference was observed regarding demographic, anthropometric, or laboratory findings. Although prolactin levels were highest in the severe group, followed by the moderate group, and lowest in the mild group, the variance between groups was statistically non-significant (P value = 0.212) (Table 3).

**Table 3:** Comparison between mild, moderate, and severe SLE patients regarding demographic, anthropometric and laboratory findings

Variables		Mild (n = 7)	Moderate (n = 18)	Severe (N = 15)	p-value
Age (years), N (%)		11.8 ± 1.0	11.3 ± 2.4	11.5 ± 2.1	^0.838
Sex, N (%)	Male	2 (28.6%)	1 (5.6%)	3 (20.0%)	§ 0.243
	Female	5 (71.4%)	17 (94.4%)	12 (80.0%)	
Consanguinity, N (%)		1 (14.3%)	8 (44.4%)	5 (33.3%)	^§ 0^.463
Family history of SLE, N (%)		1 (14.3%)	4 (22.2%)	0 (0.0%)	^§ 0^.135
Weight (SDS), mean ± SD		-0.4 ± 0.6	-0.3 ± 1.0	-0.2 ± 1.3	^^^0.910
Height (SDS), mean ± SD		-0.3 ± 0.7	-0.2 ± 1.2	-0.2 ± 1.0	^^^0.965
BMI (SDS), mean ± SD		-0.2 ± 1.4	-0.3 ± 0.7	-0.2 ± 1.2	^^^0.941
Hypertension, N (%)		6 (85.7%)	13 (72.2%)	12 (80.0%)	^§ 0^.786
Puberty features, N (%)		2 (28.6%)	11 (61.1%)	9 (60.0%)	^§ 0^.371
Platelets (x10^3^/mL), mean ± SD		286.1 ± 103.6	312.7 ± 102.2	265.8 ± 98.1	^^^0.419
ESR (mm/hr), mean ± SD		42.4 ± 17.2	33.9 ± 28.5	44.5 ± 29.2	^^^0.518
C3 (mg/dl), mean ± SD		58.1 ± 17.0	114.2 ± 65.6	95.6 ± 71.9	^^^0.149
C4 (mg/dl), mean ± SD		17.7 ± 18.1	20.4 ± 20.5	25.9 ± 26.6	^^^0.679
Positive ADNA, N (%)		4 (57.1%)	12 (66.7%)	14 (93.3%)	^§ 0^.073
Positive ANA, N (%)		6 (85.7%)	18 (100.0%)	14 (93.3%)	^§ 0^.296
Prolactin (µg/L), mean ± SD		14.0 ± 6.2	17.0 ± 5.3	19.3 ± 7.7	^^^0.212

^^^Independent t-test. ^#^Chi square test, ^§^Fisher’s Exact test

BMI: body mass index, ESR: erythrocyte sedimentation rate, ADNA: anti-double stranded DNA, ANA: antinuclear antibodies, SDS: standard deviation scores

In the present study, there was no significant variance between SLE patients with normal prolactin levels and those with hyperprolactinemia regarding age or any laboratory findings. Neurological disorders were significantly more common among SLE patients with hyperprolactinemia (P value = 0.007). Also, there was a significant difference in SLEDAI score between SLE patients with normal prolactin levels and hyperprolactinemia (P value = 0.004) (Table 4).

**Table 4:** Clinical and laboratory findings of normal and hyperprolactinemia SLE patients

		Normal prolactin (n = 32)	Hyperprolactinemia (n = 8)	P value
Age (years), mean ± SD		11.5 ± 2.2	11.5 ± 1.3	0.982
Sex, N (%)	Male Female	5 (15.6%) 27 (84.4%)	1 (12.5%) 7 (87.5%)	0.825
Malar rash, N (%)		20 (62.5%)	6 (75%)	0.507
Discoid rash, N (%)		2 (6.3%)	0	0.468
Photosensitivity, N (%)		21 (65.6%)	4 (50%)	0.414
Oral ulcer, N (%)		13 (40.6%)	3 (37.5%)	0.827
Arthritis, N (%)		20 (62.5%)	7 (87.5%)	0.177
Serositis, N (%)		7 (21.9%)	1 (12.5%)	0.553
Renal disease, N (%)		17 (53.1%)	6 (75%)	0.263
Neurological disorders, N (%)		3 (9.4%)	4 (50%)	0.007*
Hematological disorders, N (%)		15 (46.9%)	4 (50%)	0.874
Immunological abnormalities, N (%)		27 (84.4%)	6 (87.5%)	0.533
Platelets (x10^3^/ml), mean ± SD		286.5 ± 100.2	306.3 ± 107.3	0.457
ESR (cm/hr), mean ± SD		39.8 ± 25.6	27.5 ± 12.9	0.203
C3 (mg/dl), mean ± SD		22.4 ± 21.9	20.1 ± 25.7	0.509
C4 (mg/dl), mean ± SD		104.3 ± 67.6	69.8 ± 44.7	0.271
Positive ADNA, N (%)		22 (68.8%)	8 (100%)	0.068
Positive ANA, N (%)		31 (96.9%)	7 (87.5%)	0.277
SELDAI score, mean ± SD		10.9 ± 3.6	15.1 ± 3.2	0.004*

*P value < 0.05

BMI: body mass index, ESR: erythrocyte sedimentation rate, ADNA: anti-double stranded DNA, ANA: antinuclear antibodies

The different types of drugs used in treating SLE patients are shown in Table 5. There was no significant difference between SLE patients with normal prolactin levels and those with hyperprolactinemia regarding drug intake, such as steroids (P value > 0.05).

**Table 5:** Comparison between normal and hyperprolactinemia SLE patients regarding their treatment

	Normal prolactin (n = 32)	Hyperprolactinemia (n = 8)	P value
**Steroids, N (%)**	25 (78.1%)	8 (100%)	0.145
**Antimalarial, N (%)**	31 (96.9%)	8 (100%)	0.613
**Azathioprine, N (%)**	4 (12.5%)	2 (25%)	0.376
**Cyclophosphamide, N (%)**	8 (25%)	3 (37.5%)	0.479
**Cyclosporine, N (%)**	24 (75%)	7 (87.5%)	0.449
**Mycophenolate mofetil, N (%)**	11 (34.4%)	4 (50%)	0.414

## Discussion

This study revealed that hyperprolactinemia was detected in 20% of SLE cases (8/40), 2 moderate cases, and 6 severe cases. This is in line with Karimifar et al., who reported hyperprolactinemia in 8.4% (5/60) of SLE patients [21].

In the current study, prolactin levels in the SLE group showed significantly higher levels than the control group, with a P value of 0.005. Similarly, Al-Bayomy et al. and Song and Lee found that the mean prolactin level was greater in SLE patients than in controls [22, 23]. On the other hand, Soliman et al. and Jokar et al. showed no statistically significant variance in prolactin levels between SLE patients and the control group [24, 25]. Patients with SLE may develop hyperprolactinemia due to either increased pituitary prolactin secretion under the influence of inflammatory cytokines or increased synthesis of prolactin by peripheral lymphocytes [26, 27]. The PRL-anti-PRL immune complexes (macroprolactins) are not physiologically active because their large mass prevents them from passing through capillary walls and reaching their intended target regions. Increased levels of prolactin in these patients may be due to a delayed removal of the circulating PRL-IgG complex [28]. Moreover, as the prolactin gene is near the HLA complex, genetic alterations in the gene may increase the predisposition to the disease in some SLE patients [29].

After the categorization of SLE patients according to disease severity into mild, moderate, and severe groups, prolactin levels were highest in the severe group, followed by moderate and lowest in the mild group; however, the variance was statistically non-significant (P value = 0.212). Similarly, Al-Bayomy et al. found a non-significant difference between prolactin levels in both active and inactive disease groups before the start of treatment [22]. In addition, prolactin level had a significant positive correlation with the SLEDAI score, as shown in this study. Moreover, previous meta-analysis studies revealed a significantly positive correlation between SLE activity and prolactin levels [23, 30]. Several studies have found that prolactin acts as an immune stimulant and can have a direct impact on disease severity in chronic autoimmune inflammatory conditions [31]. Most immune cells release prolactin, which promotes T and B lymphocyte proliferation, differentiation, and maturation [32, 33]. The presence of higher serum prolactin levels in SLE patients, as well as higher prolactin levels in inflammatory tissue and synovial fluid with a significant relationship to disease activity, suggests that locally invaded immune cells, fibroblasts, and chondrocytes secrete prolactin in greater amounts [34]. Locally produced prolactin in inflammatory tissues stimulates the immune system and enhances it by creating more inflammatory cytokines and matrix metalloproteinases, resulting in structural alterations associated with SLE [35].

On the other hand, our results were inconsistent with the studies carried out by Soliman et al. and Jokar et al., as they showed a non-statistically significant correlation between SLEDAI score and PRL level in cases with SLE [24, 25]. The controversy and the discrepant results of the role of prolactin in SLE can be explained by many aspects, including variability of disease duration, different treatments, the heterogeneity of SLE patients enrolled, the usage of different indices to evaluate SLE activity, the circadian rhythms of prolactin, and different laboratory methods used for prolactin assay. Moreover, hyperprolactinemia is associated with auto-antibodies that may interfere with the prolactin assay [36–39].

This study also revealed a significant negative correlation between prolactin levels and C3. In agreement, Jacobi et al. found an association between high prolactin levels and indicators of disease activity, such as a decrease in complement factors and an increase in ESR [40]. In addition, Zhu et al. revealed a negative correlation between serum prolactin levels and the complement factor C3 [41].

Neurological disorders such as seizures were more prevalent among active SLE cases with hyperprolactinemia than those with normal prolactin levels in the present study (P value = 0.007). This is in harmony with Al-Garf et al., who reported that all SLE patients with hyperprolactinemia showed central nervous system (CNS) manifestations compared to only 10% of patients with normal PRL levels (P value 0.003) [42]. Also, a study done by Vera-Lastra et al. concluded that hyperprolactinemia may play a role in SLE-related CNS involvement [43]. Moreover, Pacilio et al. reported a direct relationship between hyperprolactinemia and central nervous system involvement [44]. The link between hyperprolactinemia and high IL-6 levels in neuropsychiatric lupus patients suggests that there is a reciprocal interaction between the neuroendocrine and immune systems [45].

Also, the present study revealed higher SLEDAI scores among SLE cases with hyperprolactinemia than those with normal prolactin levels (P value = 0.004). Similarly, a study done by Abdelaziz et al. found a higher SELDAI score in SLE patients with hyperprolactinemia (16.62 ± 9.14) when compared with those with normal prolactin levels (13.04 ± 6.40), but the difference was statistically insignificant [46]. Hyperprolactinemia found in patients with active SLE may be triggered by several factors [47]. One explanation is that activated lymphocytes may produce pro-inflammatory cytokines that may pass through the blood-brain barrier and trigger pituitary cells to release PRL. This concept is supported by finding PRL and interleukin 6 in the cerebral fluid of patients with active neuropsychiatric lupus [48]. Another explanation is that SLE patients have poor control of PRL secretion, as evidenced by high cyclo (His-pro), which is a PRL secretion inhibitor, and low homovanillic acid (a dopamine metabolite) [49].

In our study, there was no significant difference between SLE patients with normal prolactin levels and those with hyperprolactinemia regarding drug intake (P value > 0.05). This could indicate that high prolactin levels in SLE patients were caused by disease activity rather than drug intake, such as steroids. Similarily, Vera-Lastra et al. reported a significant correlation between SLEDAI score and prolactin levels in all SLE patients after and before medication (steroids, chloroquine, and cyclophosphamide) (r = 0.9086, p = 0.0001, and r = 0.4946, p = 0.0007, respectively) [50].

There was a female predominance, as 85% of our patients were female. In accordance, earlier studies done among Egyptian children reported a high female-to-male ratio ranging from 2.7:1 up to 12:1 [51, 52]. Considering estrogens implicated in the development of SLE, variation in pubertal conditions could be the cause of this female-to-male ratio difference among various research studies [53].

### Limitations

A small number of participants were included in our study due to financial restrictions.

## Conclusions

A relationship between serum prolactin levels and juvenile SLE disease was detected. Neurological manifestations were more prevalent among SLE patients with hyperprolactinemia.

## Data Availability

All data generated or analysed during this study are included in this published article.

## Abbreviations

ACR: American College of Rheumatology
PRL: prolactin
SLE: systemic lupus erythematosus
SELDAI: Systemic Lupus Erythematosus Disease Activity Index
ELISA: Enzyme Linked Immunosorbent Assay
PRL-R: prolactin receptor
BUN: blood urea nitrogen
AST: aspartate transaminase
ALT: alanine transaminase
CBC: complete blood count
ESR: erythrocyte sedimentation rate
ADNA: anti-double stranded DNA
ANA: anti-nuclear antibodies
SPSS: Statistical Package for Social Sciences
ANOVA: analysis of variance
r: correlation coefficient
CNS: central nervous system
SDS: standard deviation scores
